# Maf1 suppression of ATF5-dependent mitochondrial unfolded protein response contributes to rapamycin-induced radio-sensitivity in lung cancer cell line A549

**DOI:** 10.18632/aging.202584

**Published:** 2021-02-26

**Authors:** Chen Lai, Jing Zhang, Zhaohua Tan, Liang F. Shen, Rong R. Zhou, Ying Y. Zhang

**Affiliations:** 1 Department of General Surgery, Xiangya Hospital of Central South University, Changsha 410008, Hunan, China; 2 Hunan Key Laboratory of Precise Diagnosis and Treatment of Gastrointestinal Tumor, Changsha 410008, Hunan, China; 3 Department of Oncology, Xiangya Hospital of Central South University, Changsha 410008, Hunan, China; 4 Xiangya Lung Cancer Center, Xiangya Hospital, Central South University, Changsha 410008, Hunan, China

**Keywords:** radio-resistance, non-small cell lung cancer cell, mitochondrial unfolded protein response, mTOR, Maf1

## Abstract

mTOR is well known to promote tumor growth but its roles in enhancing chemotherapy and radiotherapy have not been well studied. mTOR inhibition by rapamycin can sensitize cancer cells to radiotherapy. Here we show that Maf1 is required for rapamycin to increase radio-sensitivity in A549 lung cancer cells. In response to ionizing radiation (IR), Maf1 is inhibited by Akt-dependent re-phosphorylation, which activates mitochondrial unfolded protein response (UPR^mt^) through ATF5. Rapamycin suppresses IR-induced Maf1 re-phosphorylation and UPR^mt^ activation in A549 cells, resulting in increased sensitivity to IR-mediated cytotoxicity. Consistently, Maf1 knockdown activates ATF5-transcription of mtHSP70 and HSP60, enhances mitochondrial membrane potential, reduces intracellular ROS levels and dampens rapamycin’s effect on increasing IR-mediated cytotoxicity. In addition, Maf1 overexpression suppresses ethidium bromide-induced UPR^mt^ and enhances IR-mediated cytotoxicity. Supporting our cell-based studies, elevated expression of UPR^mt^ makers (mtHSP70 and HSP60) are associated with poor prognosis in patients with lung adenocarcinoma (LAUD). Together, our study reveals a novel role of Maf1-UPR^mt^ axis in mediating rapamycin’s enhancing effect on IR sensitivity in A549 lung cancer cells.

## INTRODUCTION

Rapamycin and its analogs (rapalogs) inhibit cell growth, which is a key mechanism underlying its effectiveness in cancer treatment [1, 2]. Recently, rapamycin has also been shown to improve radiotherapy and chemotherapy in some cancers, including lung cancer [3–5]. Biochemically, rapamycin, along with FKBP12, binds and inhibits a protein kinase called mTOR in a highly specific manner [1]. There are two mTOR complexes in human cells, mTORC1 and mTORC2, defined by different associated proteins. Only mTORC1 is sensitive to rapamycin [6]. Different from many other kinase inhibitors, rapamycin does not directly inhibit the ATP catalytic activity of mTORC1 [1]. Therefore, mTORC1 activity is commonly evaluated through the phosphorylation status of mTOR kinase substrates S6K1, 4E-BP1 and Maf1 [7, 8]. These effectors mediate diverse biological processes including ribosome biogenesis, autophagy, immunity and aging [9–12].

Akt is a protein kinase lies upstream of mTORC1 to mediate nutrient and insulin regulation of cell growth [1, 2]. Akt is mutated in many types of cancer cells and has been linked to radiotherapy and chemotherapy failure [13, 14]. In response to ionizing radiation (IR), Akt is activated through phosphorylation at Serine-437 by PI3K (Phosphoinositide 3-kinase), therefore contributing to radio-resistance [15–17]. Deregulation of the mTOR effectors S6K1/2 and 4E-BP1 has also been linked to IR resistance [15, 18, 19].

Maf1 is an mTORC1 effector that has significant roles in cancer biology [20–24]. Maf1 is phosphorylated by mTORC1 at certain Serine and Threonine to regulate RNA Polymerase III-dependent transcription of tRNAs, microRNAs and other small nuclear RNAs [7, 25, 26]. Inhibiting Maf1 expression can induce cancerous transformation in hepatocellular cells and Maf1 overexpression can suppress tumor growth *in vivo* [21, 23]. Although unclear on the mechanisms, Maf1 has been shown to regulate mitochondrial morphology and functions and loss of Maf1 in *C. elegans* activates UPR^mt^ [27].

UPR^mt^ is a signaling pathway communicating between the nuclear transcription and mitochondrial proteostasis [28, 29]. Loss of mitochondrial protein homeostasis induces nuclear transcription of mitochondrial chaperones such as HSP60 mtHSP70, which helps restore the normal mitochondrial functions. Recently, the transcription factor ATF5 was identified to mediate transcription of UPR^mt^ target genes [30]. Treating cells with DNA intercalator ethidium bromide (EtBr) depletes mitochondrial DNA and activates UPR^mt^ in a ATF5-dependent manner [30]. ATF5 overexpression promotes radio-resistance in lung cancer cells [31]. Aberrant UPR^mt^ signaling is implicated in multiple diseases, such as cancers and neurodegenerative diseases [29, 32].

As a well-known mTOR effector, Maf1’s role in cancer radiosensitivity has not been investigated. In this study, we find that Maf1 is required for rapamycin to sensitize A549 lung adenocarcinoma cells to X-ray. Maf1 is inhibited through AKT-dependent re-phosphorylation in response to X-ray. Adding rapamycin under this condition activates Maf1 and enhances IR-mediated cytotoxicity. In addition, we find that Maf1 functions to suppress UPR^mt^, a pro-survival mechanism in response to IR. Maf1 knockdown dampens rapamycin’s enhancing effect on IR toxicity and Maf1 overexpression is sufficient to increase IR toxicity to A549 cells. Our study implicates Maf1 regulation of UPR^mt^ as a novel mechanism mediating rapamycin’s enhancing effect on radiosensitivity of lung cancer cells.

## RESULTS

### Maf1 is required for rapamycin to increase radio-sensitivity in A549 cells

**Figure 1:**
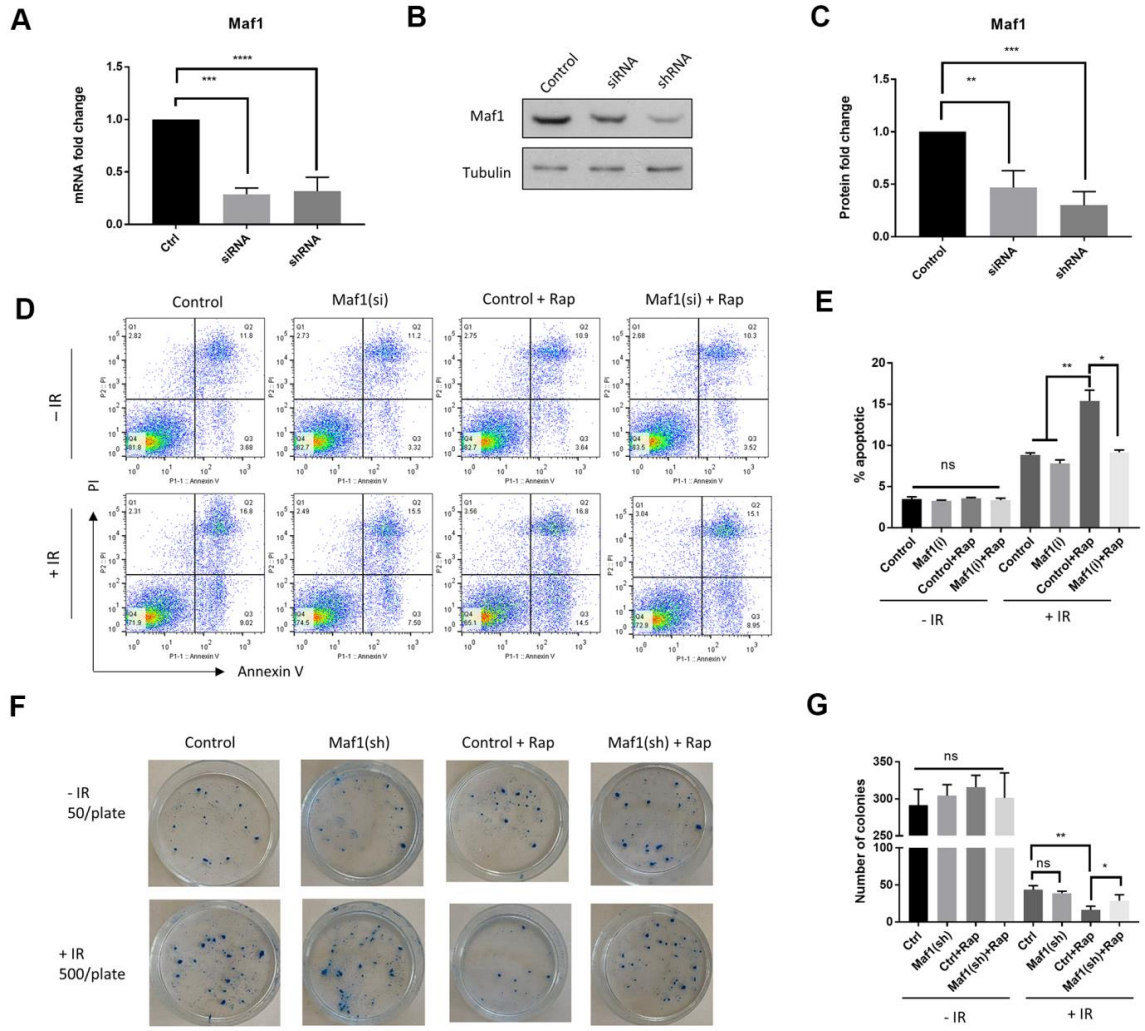
**Maf1 is required for rapamycin to increase radio-sensitivity in A549 cells.** (**A**–**C**) Maf1 mRNA (**A**) and protein levels (**C**) were knocked down by siRNA and shRNA. Representative western blot results are shown in (**B**). Experiments were performed for ≥3 times with replicates. Data were normalized to non-transfected control (Ctrl) and expressed as fold change. (**D**, **E**) Maf1 is required for rapamycin to increase apoptosis in A549 cells in response to radiation. Maf1 was knocked down by siRNA (si) in A549 cells. Cells were then irradiated (IR) with 6 Gy x-ray and treated with 100 nM rapamycin (Rap) as indicated. Apoptosis (Annexin V) and cell death (PI) were analyzed by flow cytometry after 48 hours. Experiments were performed for 2 times and representative results (**D**) and the quantifications of apoptotic cells (**E**) are shown. (**F**, **G**) Maf1 is required for rapamycin to enhance radiosensitivity in A549 cells. Cells treated with radiation and rapamycin were plated at 500 cells/plate and irradiated (+IR). Non-irradiated cells were plated at 50 cells/plate as controls (-IR). Colonies were counted after 2 weeks of incubation. Experiments were performed for 3 times and representative results are shown in (**F**) and the quantifications in (**G**). In all panels, the error bars stand for Standard Deviation (SD) of the mean. Statistical significance was evaluated by 2-tailed, paired student’s t-test (ns, not significant, *, P<0.05, **, P<0.01, ***, P<0.001, ****, P<0.0001).

Rapamycin inhibition of mTOR kinase sensitizes cancer cells to radiotherapy [3, 17]. The mTOR effector protein S6K1 plays a key role in chemo-resistance [17]. We wondered if Maf1, a poorly studied mTOR effector could also have roles in radio-resistance in cancer cells. To this end, we knocked down Maf1 expression in A549 cells by siRNA. Both siRNAs and shRNA used in this study effectively reduced Maf1 expression as determined by qPCR and western blot (Figure 1A–1C). To determine the effect of Maf1-knockdown on radio-sensitivity, we first examined cell death and apoptosis through propidium iodide (PI) and Annexin V respectively, followed by flow cytometry. A549 cells were first transfected with Maf1 siRNA or vehicle controls for 48 hours then exposed to 6 Gy of X-ray. Cells were then treated with 100 nM rapamycin for 48 hours. Our results showed that rapamycin significantly increased the percentage of apoptotic cells treated with IR. Interestingly, Maf1 knockdown almost blocked rapamycin’s effect (Figure 1D, 1E). Maf1 knockdown alone did not significantly alter the percentage of apoptotic cells, suggesting that Maf1’s effect was specific to rapamycin. Without IR, rapamycin did not significantly increase apoptotic population in our hands. Neither Maf1 knockdown nor 24-hour rapamycin treatment significantly increased PI-stained populations (Figure 1D). We also examined the effect of Maf1 knockdown on rapamycin-mediated cell proliferation. Rapamycin robustly reduced cell proliferation in a dose-dependent manner (Supplementary Figure 1). However, rapamycin similarly decreased cell proliferation in A549 cells regardless of Maf1 knockdown. In the presence of IR, rapamycin at 100 nM also decreased proliferation (Supplementary Figure 2). Therefore, Maf1 was not involved in rapamycin regulation of A549 cell proliferation.

We next examined the role of Maf1 on IR-induced cytotoxicity by clonogenic assay. A549 cells with or without Maf1 knockdown were irradiated, then treated with 100 nM rapamycin. Cells were allowed to grow for 2 weeks to form colonies. Consistent with a previous study [3], rapamycin enhanced the IR-induced cytotoxicity. Interestingly, such enhancing effect was mostly blocked by Maf1 knockdown (Figure 1F, 1G). Maf1 knockdown alone did not appear to affect the clonogenicity, indicating that Maf1 was specifically required for rapamycin to sensitize A549 cells to X-ray.

### Rapamycin suppresses Akt-mediated Maf1 re-phosphorylation in response to IR in A549 cells

**Figure 2:**
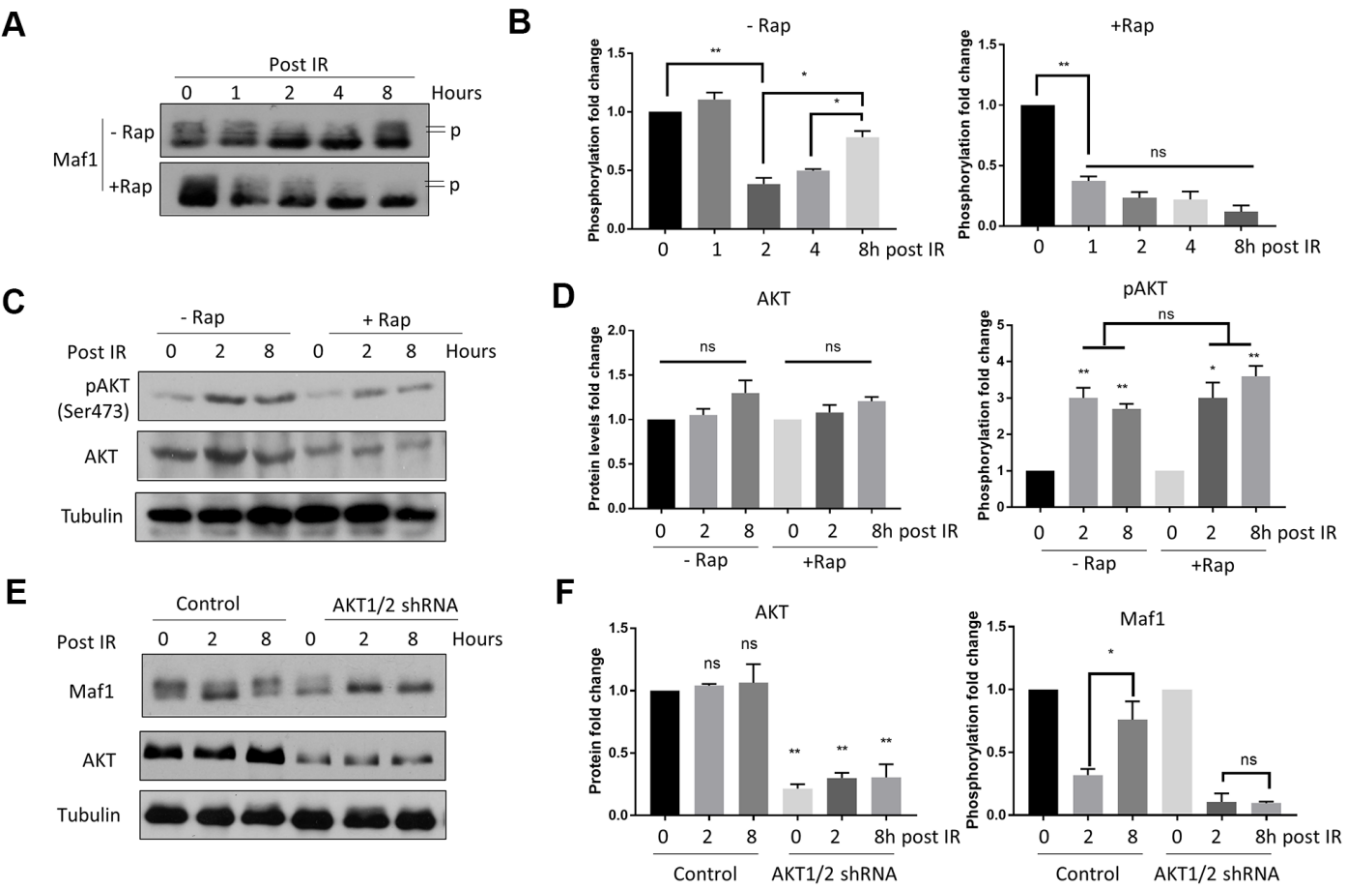
**Rapamycin suppresses Akt-mediated Maf1 re-phosphorylation in response to IR in A549 cells.** (**A**, **B**) Maf1 phosphorylation (P) was decreased by IR but was re-phosphorylated after 8 hours (Post IR). Rapamycin inhibited Maf1 re-phosphorylation. A549 cells were irradiated (IR) at 6 Gy then treated with 100 nM rapamycin (Rap) for 48 hours. Maf1 phosphorylation was detected by slow migration by Western blot. (**C**, **D**) Akt phosphorylation at Ser473 was increased by IR. Akt phosphorylation was detected with phospho-specific antibody. (**E, F**) Akt was required for Maf1 re-phosphorylation in response to IR. A549 cells with stable Akt knockdown were analyzed by Western blot at indicated time points. In all panels, representative data and the quantifications of at least 2 biological repeats are shown. Data were normalized to time 0. The error bars stand for Standard Deviation (SD) of the mean. Statistical significance was evaluated by 2-tailed, paired student’s t-test (ns, not significant, *, P<0.05, **, P<0.01).

Maf1 is a well-known transcription factor responsive to diverse signals including DNA damage [25, 33, 34]. Since IR can cause DNA damage, we asked if Maf1 phosphorylation was changed in response to IR. Maf1 was phosphorylated at multiple sites by mTOR, causing slower migration on acrylamide gel, which can be detected by western blot [7]. We found that IR rapidly decreased Maf1 phosphorylation, but Maf1 phosphorylation was rapidly recovered 8 hours post-IR (Figure 2A). Such re-phosphorylation was dependent on mTOR, as rapamycin treatment blocked Maf1 re-phosphorylation (Figure 2A–2C).

Akt can be activated by phosphorylation in response to IR, which contributes to radio-resistance [15]. We found that AKT phosphorylation was increased 4 hours after 6 Gy IR (Figure 2C, 2D). Since Akt is the upstream kinase activating mTOR, we tested if Akt could contribute to Maf1 re-phosphorylation in response to IR. Indeed, knocking down Akt1/2 via shRNA blocked Maf1 re-phosphorylation (Figure 2E, 2F). These experiments suggest that AKT-dependent re-phosphorylation of Maf1 could be a potential mechanism for radio-resistance in A549 cells and that rapamycin could sensitize A549 cells to IR by inhibiting Akt-mediated Maf1 re-phosphorylation.

### Rapamycin suppresses IR-induced mitochondrial UPR through Maf1 in A549 cells

**Figure 3:**
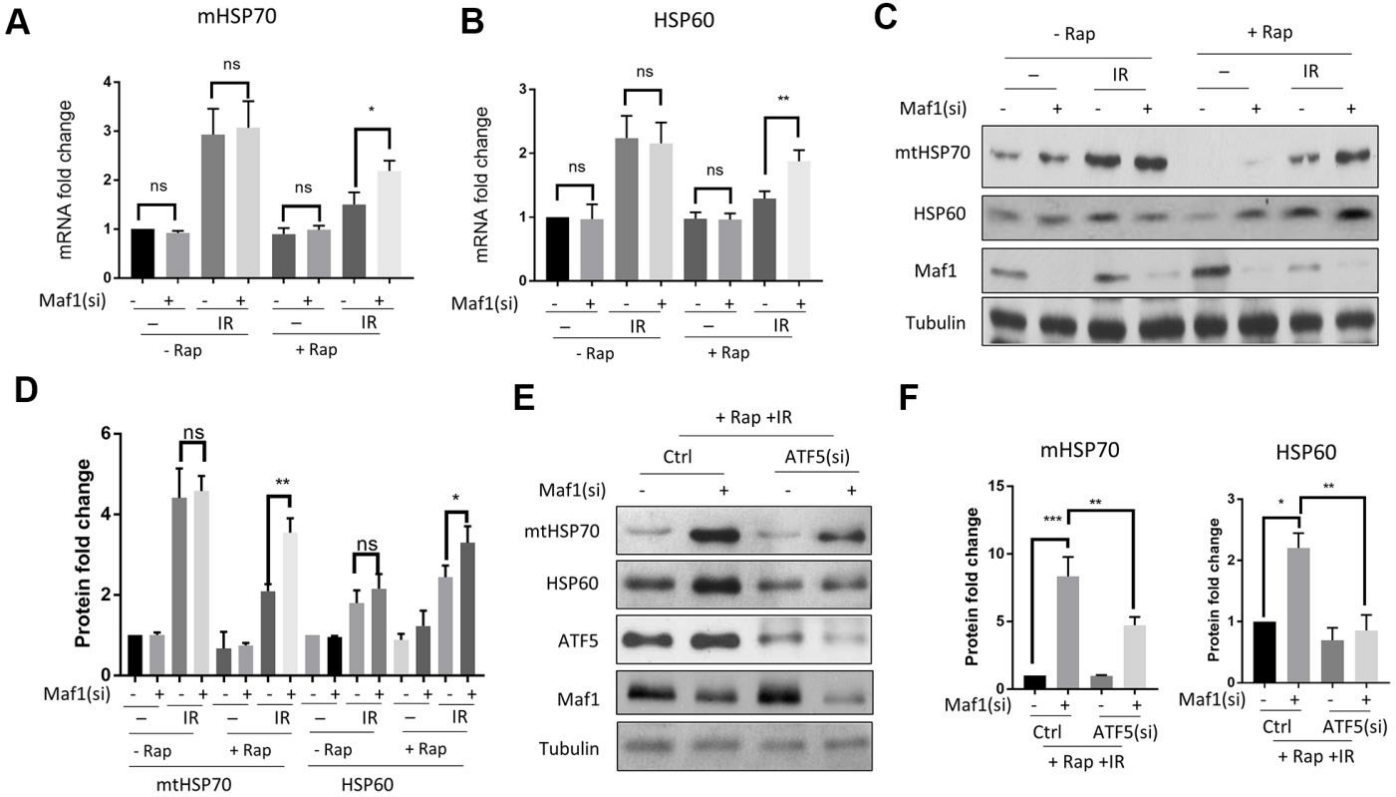
**Rapamycin suppresses IR-induced UPR**^mt^ **through Maf1 in A549 cells.** (**A**, **B**) A549 cells treated with IR and rapamycin were examined for UPR^mt^ marker genes (mHSP70 and HSP60) expression by RT-qPCR. Experiments were performed for 3 biological repeats and data were normalized to non-treated control. (**C**, **D**) HSP70 and HSP60 protein levels were analyzed by Western blot. Representative results are shown in (**C**) and quantification of 3 biological repeats in (**D**). Data were normalized to non-treated control. (**E**, **F**) Maf1 knockdown activated UPR^mt^ in an ATF5-dependent manner. A549 cells were knocked down for Maf1, ATF5 or both, then treated with rapamycin and IR. mHSP70 and HSP60 protein levels were analyzed by Western blot. Representative results are shown in (**E**). Experiment were performed for 3 times. Data were normalized to non-transfected control. For all bar graph, the error bars stand for Standard Deviation (SD) of the mean. Statistical significance was evaluated by 2-tailed, paired student’s t-test (ns, not significant, *, P<0.05, **, P<0.01, ***, P<0.001).

Maf1 was recently reported to inhibit mitochondrial UPR (UPR^mt^) in the round worm *C. elegans* [27]. We tested if IR inhibition of Maf1 in A549 cells could activate UPR^mt^. By examining UPR^mt^ markers before and 8 hours after IR, we found that IR robustly induced mtHSP70 and HSP60 mRNA expression (Figure 3A, 3B). The up-regulation of these UPR^mt^ markers was repressed by rapamycin in a Maf1-dependent manner (Figure 3A, 3B). The role of Maf1 in UPR^mt^ were further confirmed by Western blot analysis of mtHSP70 and HSP60 protein levels (Figure 3C, 3D). Maf1 knockdown did not appear to significantly increase UPR^mt^ without IR or rapamycin treatment, as judged by both mRNA and protein levels of mtHSP70 and HSP60 (Figure 3A–3D). These results suggest that Maf1 was activated by rapamycin to inhibit UPR^mt^ in response to X-ray.

Second, we tested if Maf1 regulated UPR^mt^ through ATF5, a transcription factor that was recently implicated in UPR^mt^ [30]. Although the role of UPR^mt^ in radio-resistance in cancer cells remains unclear, ATF5 has been known to increase radio-resistance in some cancer cells [31, 35, 36]. By siRNA knocking down ATF5 and Maf1, and treating cells with IR or rapamycin, we found that ATF5 knockdown significantly reduced HSP60 and mtHSP70 expression due to Maf1 loss (Figure 3E, 3F), suggesting that ATF5 was required for Maf1 to suppress UPR^mt^.

### Maf1 mediates rapamycin inhibition of mitochondrial function in response to IR in A549 cells

**Figure 4:**
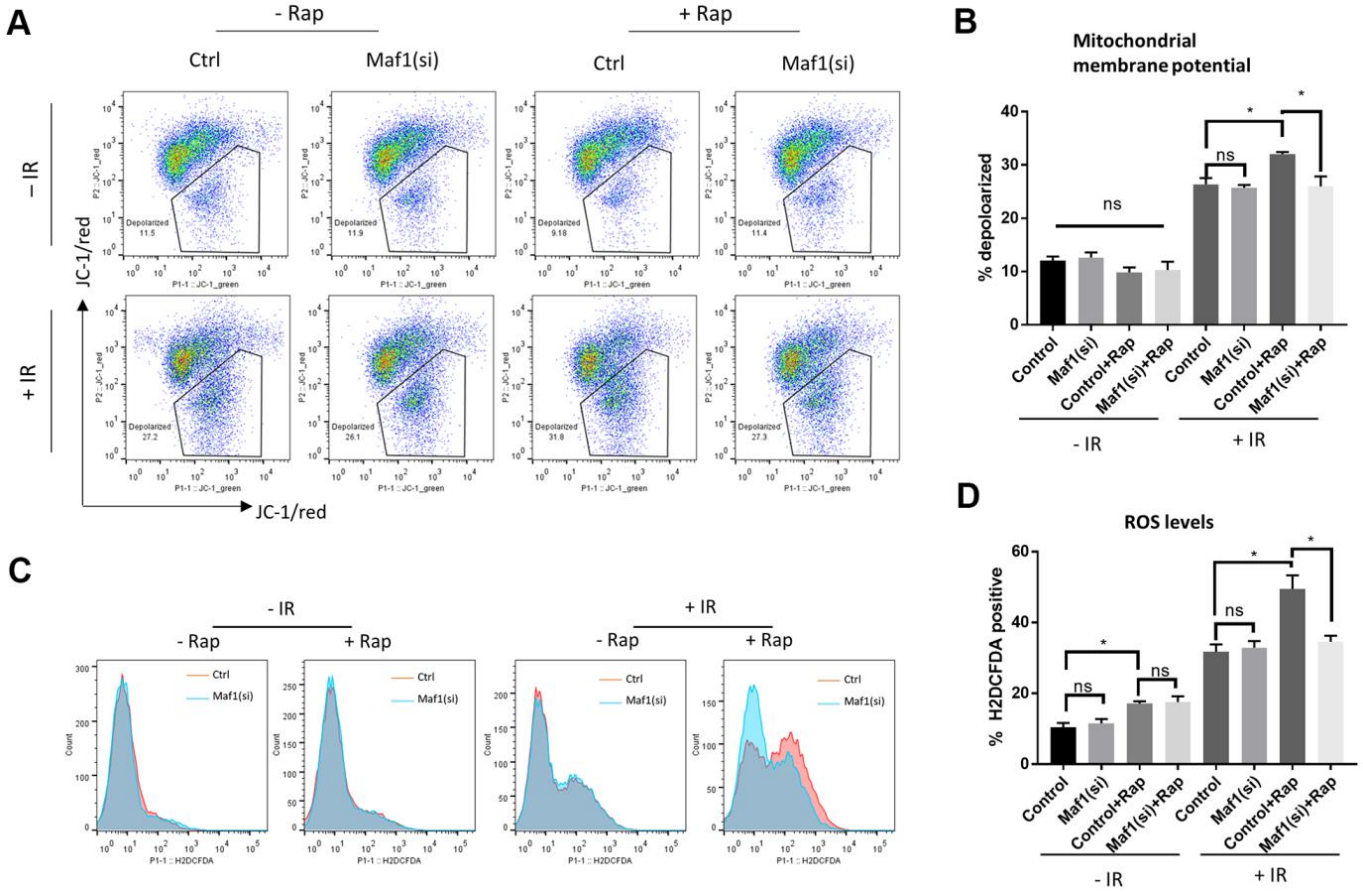
**Maf1 knockdown mitigates rapamycin inhibition of mitochondrial function in response to IR in A549 cells.** (**A**, **B**) Mitochondrial membrane potential was evaluated by JC-1 staining followed by flow cytometry. Representative results are shown in (**A**) and the quantifications of 2 biological replicates are shown in (**B**). (**C**, **D**) Intracellular ROS levels were detected by H2DCFCA probes, followed by flow cytometry. Representative results are shown in (**C**) and the quantifications of 2 biological replicates are shown in (**D**). For all bar graph, the error bars stand for Standard Deviation (SD) of the mean. Statistical significance was evaluated by 2-tailed, paired student’s t-test (ns, not significant, *, P<0.05).

UPR^mt^ can promote cell survival through improving mitochondrial function. Since Maf1 suppressed UPR^mt^ in rapamycin-treated A549 cells, we asked if Maf1 was involved in regulating mitochondrial functions. We first examined mitochondrial membrane potential (Δψ_m_) by JC-1 staining and flow cytometry. Maf1 was knocked down by siRNA then A549 cells were treated with and without IR or rapamycin as indicted in Figure 4A. The result showed that rapamycin treatment of non-irradiated cells slightly increased Δψ_m_, however, rapamycin treatment of irradiated cells exacerbated Δψ_m_ depolarization by IR, which was largely rescued by Maf1 knockdown. Maf1 knockdown did not appear to affect cells without rapamycin treatment or without IR, suggesting that Maf1 regulation of membrane potential was specific to IR and activated by rapamycin.

We next examined the generation of reactive oxygen species (ROS). The increase in ROS levels is a hallmark of impaired mitochondrial function, which could trigger apoptosis and cell death. We stained cells with a widely used ROS dye H2DCFDA and analyzed ROS levels through flow cytometry. Consistent with JC-1 data (Figure 4A, 4B), rapamycin enhanced IR to generate toxic ROS levels, which could be reversed by Maf1 knockdown (Figure 4C, 4D). Rapamycin slightly increased ROS levels in non-irradiated cells but such increase did not appear to be affected by Maf1 knockdown. In addition, Maf1 knockdown did not affect ROS levels in cells treated with rapamycin or IR alone, suggesting a specific role of Maf1 in the interface.

### Maf1 overexpression suppresses UPR^mt^ and sensitizes A549 cells to IR

**Figure 5:**
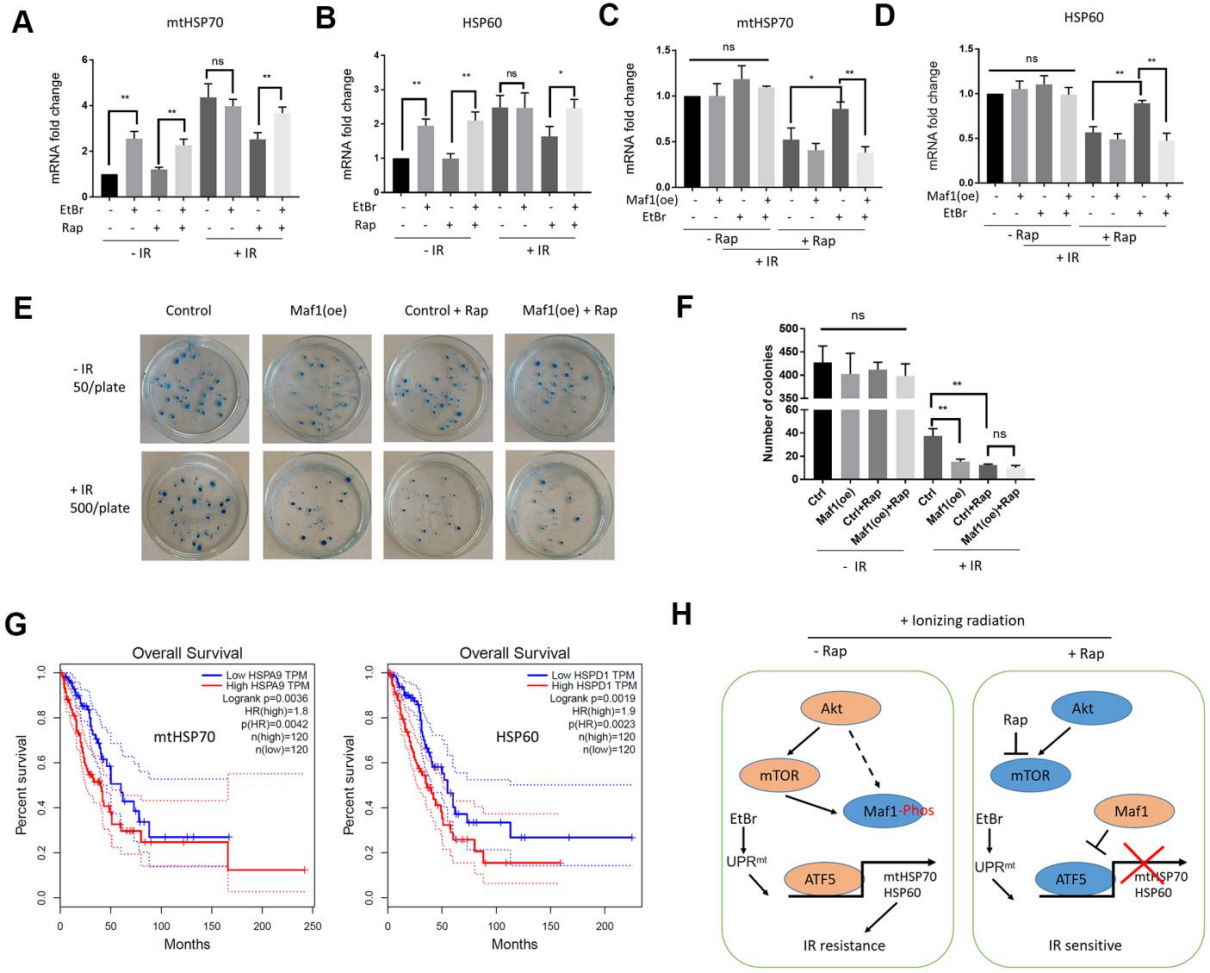
**Maf1 overexpression suppresses UPR**^mt^**-induced IR resistance in A549 cells.** (**A**, **B**) EtBr activates UPR^mt^ in the presence of rapamycin. A549 cells were treated with indicated drugs or/and IR. UPR^mt^ marker genes (HSP60 and mtHSP70) were examined by RT-qPCR. (**C**, **D**) Maf1 overexpression (oe) prevents EtBr from increasing HSP60 and mtHSP70 expression in the present of rapamycin and IR. (**E**, **F**) Maf1(oe) sensitizes A549 cells to IR and is not additive to rapamycin. A549 cells (500 cells/plate) stably expressed Maf1 were treated with rapamycin and IR, then allowed to form colonies for 2 weeks. A549 cells were plated at 50 cells/plate as non-irradiated controls. Representative data are shown in (**E**) and the quantifications of 3 biological replicates in (**F**). (**G**) High expression of UPR^mt^ marker genes (HSP60 and mtHSP70) are significantly associated with poor prognosis in lung adenocarcinoma (LAUD) patients. (**H**) A working model showing the role of Maf1-UPR^mt^ in mediating rapamycin’s enhancing effect on IR sensitivity. For all bar graph, the error bars stand for Standard Deviation (SD) of the mean. Statistical significance was evaluated by 2-tailed, paired student’s t-test (ns, not significant, *, P<0.05, **, P<0.01).

To test the effect of Maf1 overexpression on UPR^mt^, both Maf1 and UPR^mt^ need to be activated. EtBr is a DNA intercalating agent known to induce UPR^mt^ [30], and rapamycin can activate Maf1. Pretreating A549 cells with EtBr and rapamycin robustly increased mtHSP70 and HSP60 mRNA levels (Figure 5A, 5B). Importantly, Maf1 overexpression under such conditions indeed suppressed mtHSP70 and HSP60 expression (Figure 5C, 5D), confirming the negative role of Maf1 in UPR^mt^ regulation.

Next, we evaluated the effect of Maf1 overexpression on IR resistance by clonogenic assay. Consistently, Maf1 overexpression sensitized A549 cells to IR (Figure 5E, 5F). Maf1 overexpression did not further enhance rapamycin’s effect on IR toxicity, suggesting the specific effect of Maf1 in mediating rapamycin regulation of IR sensitivity. Interestingly, Maf1 overexpression did not appear to affect clonogenicity of A549 cells without IR, suggesting that Maf1 was largely inactivated in A549 cells under normal growing conditions.

### UPR^mt^ markers mtHsp70 and HSP60 are associated with poor prognosis in lung adenocarcinoma (LAUD) patients

We wondered if UPR^mt^ markers involved in rapamycin regulation of IR sensitivity could be important in LAUD patients. Although there have been several studies showing the prognostic values of mtHSP70 and HSP60 in lung adenocarcinoma patients [37, 38], they usually analyzed in-house data. We analyzed a huge RNAseq dataset originally from NIH TGCA database by using a recently developed algorithm at http://gepia.cancer-pku.cn/ website [39]. Based on the RNAseq data, 240 patients were grouped to 120 mtHSP70-high group and 120 mtHSP70-low group. By plotting the Kaplan Meier curve, we showed that mtHSP70-high patients had significantly worse survival (Figure 5G). The hazard rate was 1.8, indicating that LAUD patients with higher mtHSP70 expression were 1.8 times more likely to die from cancer than patients expressing lower mtHSP70. Similar results were obtained for patients with high HSP60 expression (Figure 5G). These results suggest that elevated UPR^mt^ is a poor prognosis factor for LAUD patients, consistent with the roles of UPR^mt^ in mediating Maf1 regulation of IR toxicity in A549 lung cancer cells.

## DISCUSSION

In this study, we for the first time implicate Maf1, an effector of mTOR in regulating IR sensitivity. The results suggest that genetically or pharmacologically activating Maf1 could improve radiotherapy for lung cancers or even other cancers, especially those that are resistant to rapamycin or deregulated in mTOR activity. We propose that Maf1 increases IR sensitivity through repressing ATF5-dependent UPR^mt^. In the absence of rapamycin, UPR^mt^ is activated by IR, which could serve as a feedback mechanism to promote survival, and Maf1 is phosphorylated and inhibited by AKT- and/or mTOR. Upon rapamycin treatment, Maf1 is activated through dephosphorylation and represses ATF5-dependent transcription of UPR^mt^ genes, leading to increased IR sensitivity (Figure 5H). Note that our study has been conducted in only one single cell line (A549). It will be important to test whether such mode of regulation remains true in other cancer cells.

The kinase regulating Maf1 in response to IR remains to be clarified. Maf1 has been known to be dephosphorylated and activated by DNA damage agent [7]. Consistently, our results show that Maf1 is rapidly dephosphorylated by IR. Interestingly, we found that Maf1 phosphorylation was recovered 8 hours after IR treatment. IR has been known to increase Akt phosphorylation and the Akt homolog Sch9 in yeast cells can directly phosphorylate Maf1 [15, 40]. In A549 cells however, Akt is not likely a direct kinase to Maf1 as we did not observe interaction or phosphorylation *in vitro* (data not shown). In addition, no conserved Akt phosphorylation motifs were found on Maf1 protein. Alternatively, mTOR could be the direct kinase as mTOR has been shown to phosphorylate Maf1 directly and in current study, the re-phosphorylation of Maf1 is rapamycin-dependent. In addition, Maf1 knockdown did not affect apoptosis or clonogenicity in the absence of rapamycin, supporting a direct role of mTOR in the regulation of Maf1 for radiosensitivity.

How Maf1 represses UPR^mt^ remains unclear. Maf1 was initially identified in a screen to regulate mitochondrial localization of a tRNA modifying enzyme Mod5 [41]. Loss of yeast Maf1 inhibits cell growth in nonfermentable carbon source [42], suggesting a role of Maf1 in regulating mitochondrial function. In a recent study in *C. elegans*, UPR^mt^ was found to be activated in Maf1 mutant [27]. Our results in A549 cells suggest similar mode of regulation: Maf1 knockdown activates the transcription of UPR^mt^ marker genes HSP60 and mtHSP70. In addition, we further implicate the transcription factor ATF5 in mediating Maf1 regulation of UPR^mt^. Maf1 inhibits Polymerase III (Pol III)-dependent transcription of tRNA and microRNA. One scenario is that certain Maf1-regulated microRNAs binds and repress the transcription of UPR^mt^ regulators to regulate IR sensitivity, as there are already many studies implicating tRNA and microRNA in the regulation of IR sensitivity. Alternatively, Maf1 could regulate UPR^mt^ through modulating mitochondrial functions to indirectly antagonize UPR^mt^. Note that pathways other than UPR^mt^ could also participate and play more important roles in the regulation of IR sensitivity by Maf1. It will be interesting to conduct a systematic analysis of many IR resistance-related pathways by using RNAseq or genome-wide siRNA screen.

Our analysis of publicly available database lends further support to the role of UPR^mt^ in the regulation of IR sensitivity. By examining the RNAseq and survival data from 240 patients with LAUD deposited in NIH TGCA database, we show that high expression of both HSP60 and mtHSP70 are correlated with poor prognosis in LAUD patients, suggesting an important role of UPR^mt^ in cancer progression. Several previous papers also report similar results in other types of cancer regarding mtHSP70 and HSP60 [37, 43–45]. However, our study here indicates that UPR^mt^ rather than some individual chaperones is involved in IR resistance in cancer cells. Further characterization of UPR^mt^ in cancer therapy resistance could open new window for better treatment strategies. Maf1 and ATF5 have no prognostic values for LAUD, likely due to the fact that they are usually regulated through posttranslational modifications instead of protein abundance.

Rapamycin-induced autophagy has been shown to sensitize A549 cells to IR [3]. As an effector downstream of mTOR, Maf1 is activated by rapamycin to inhibit Pol III-dependent transcription. In this sense, Maf1 loss should prevent rapamycin from inducing autophagy, therefore increasing IR resistance. This idea is consistent with our results. However, contrast to the positive role of Maf1 in autophagy, Maf1 knockout in mice and *C. elegans* induces autophagy [27, 46]. In this scenario, Maf1 knockdown should sensitize A549 cells to IR, which is contradictory to our data. In addition, literature regarding the role of autophagy in IR sensitivity remains inconsistent [47]. Therefore, whether autophagy is involved and how it modulates IR sensitivity in the context of Maf1-UPR^mt^ remains to be investigated.

## MATERIALS AND METHODS

### Irradiation and drug treatment

HEK293 and A549 cells were originally obtained from The Cell Bank of Type Culture Collection of Chinese Academy of Sciences. A549 cells were maintained in DMEM supplemented with 2 % (vol/vol) FBS. Cells were first transfected with siRNA to knockdown gene expression for 48 hours where necessary, then exposed to 6 Gy of X-ray radiation. Cells were then immediately treated with or without 100 nM rapamycin and maintain for 48 hours. For EtBr treatment, cells were treated with 500 ng/mL EtBr for 4 days to deplete mitochondrial DNA before irradiation.

### siRNA and shRNA knockdown and overexpression

Maf1 siRNA (5’-GGCUCAAGCGAAUCGUCUUTT-3’) and ATF5 siRNA (5’-GTCCAAATCATGAAATGTTTG-3’) published before [20, 48]. For transient knockdown, cells were transfected with siRNA using siRNA-Mate transfection reagent (Genepharma) according to manufacturer’s instruction. Validated Maf1 shRNA, Akt1 shRNA and Akt2 shRNA in pLKO.1 plasmid were purchased from Millipore. Lentiviral particles were prepared by transfecting HEK-293T cells with shRNA plasmid and packing plasmids psPAX2 and pMD2.G. Lentiviral particles were used to infect A549 cells and select for stable cell line with puromycin. For Maf1 overexpression, Maf1 gene coding region was cloned under the CMV promoter in pcDNA3 vector. A549 cells were transfected with pcDNA3-Maf1 plasmid by using Lipofectamine 2000 and selected for stable cell line with G418.

### Real-time quantitative PCR

A549 cells were washed with PBS for 3 times and directly lysed in TRIzol Reagent (Invitrogen). Samples were mixed with chloroform to separate protein from RNA. Aqueous phase was transferred to new tubes and containing RNA were precipitated with isopropanol. RNA was washed with 70% ethanol and suspended in RNase-free H_2_O. RNA was reverse transcribed by using HiScript II Q RT SuperMix for qPCR (Vazyme) according to provider’s manual. RT-qPCR was performed in T100 Thermal Cycler (Bio-Rad) using AceQ Universal SYBR qPCR Master Mix (Vazyme). Primers sets are published before [20, 30] and as follows: MAF1, 5'-ctcacagctgactgtggagact-3', 5'-aacatgtgtttgtcgtctcctg-3'; GAPDH (internal control), 5'-agccacatcgctcagacac-3', 5'-gcccaatacgaccaaatcc-3'; HSP60, 5’-GATGCTGTGGCCGTTACAATG-3’, 5’-GTCAATTGACTTTGCAACAGTCACAC-3’; mtHSP70, 5’-CAAGCGACAGGCTGTCACCAAC-3’, 5’-CAACCCAGGCATCACCATTGG-3’.

### Western blotting

Cells attached to the culture dish were washed with room temperature PBS for 3 times, then lysed with equal volume of 2X SDS-PAGE sample loading buffer (diluted from 4X loading buffer (250 mM Tris-HCl pH 6.8; 8 % SDS; 0.2 % Bromophenol Blue; 20% β-mercaptoethanol; 40 % glycerol)) by repeated pipetting. Whole cell lysates were heated at 95° C for 5 min and separated with SDS-PAGE and transferred to PVDF membrane. Membranes were blocked in 5% non-fat milk then probed with primary antibodies in 5% non-fat milk. Membranes were washed with PBST (PBS+0.5% Tween-20) then incubated with HRP-conjugated secondary antibodies. After extensive wash with PBST, bound HRP-conjugated secondary antibody was detected by ECL. Primary antibodies: MAF1 Polyclonal Antibody (Invitrogen), Akt and Phospho-Akt (Ser473) Antibody (Cell Signaling), ATF5 Polyclonal Antibody (Invitrogen), β-Tubulin (Cell Signaling).

### Flow cytometry

Apoptosis was measured by staining cells with Annexin V. Cells were washed with ice cold PBS and stained with biotinylated Annexin V (Biolegend) at suggested concentration for 15 min, washed, then incubated with FITC-conjugated streptavidin (Biolegend). Cells were washed with PBS extensively to remove non-specific binding. Membrane potential was measured by using JC-1 dye (ThermoFisher) as described in the product manual. Briefly, cells in DMEM medium were incubated with an equal volume of staining solution containing 5μg/ml JC-1 at 37° C for 20 min. Cells were washed for 3 times with PBS and resuspended in DMEM. ROS was measured by staining PBS-washed cells with 10 μM DCHF-DA at 37° C for 30 min in the dark. Cells were then washed with PBS and resuspended in DMEM. Flow cytometry analysis was performed on cytoFLEX S (BECKMAN). Data were analyzed with FlowJo V10.7.

### Clonogenic assay

A549 cells were plated on 35mm poly-D-lysine-treated cell culture dish in triplicates to obtain 50 cells/well (control) or 500 cell/well (irradiated). Overnight cultured were then exposed to 6 Gy of radiation in Siemens MD2 Linear Accelerator. Cells were then treated with or without rapamycin for 48 hours. Fresh DMEM medium without rapamycin were used to grow colonies for 14 days. After 2 weeks of culture, cells were washed with PBS and stained with a mixture of 0.5% crystal violet in 50/50 methanol/water for 30 min. Plates were gently rinsed with water and dried at room temperature. Plate were imaged and colonies were counted by eyes.

## Supplementary Material

Supplementary Methods

Supplementary Figures
