# Supplementary material for: Maf1 suppression of ATF5-dependent mitochondrial unfolded protein response contributes to rapamycin-induced radio-sensitivity in lung cancer cell line A549
Source: Aging (Albany NY). 2021 Feb 26;13(5):7300–13. doi: 10.18632/aging.202584 (PMC7993702; doi:10.18632/aging.202584)
Supplement: Supplementary Methods [file aging-13-202584-s001.pdf]

SUPPLEMENTARY MATERIALS

Supplemental Methods

MTT viability assay

MTT Cell Growth Assay Kit (CT02) was purchased from Millipore and experiment was conducted according to manufacturer's protocol. Briefly, A549 cells were cultured at 96-well plate as indicated by different experiments. MTT reagent was equilibrated in PBS buffer at room temperature to 5 mg/ml before use. Cells were removed of medium and 20 μl MTT working solution was added to each well, then incubated at 37° C for 4 hours. The MTT solution was carefully removed by aspiration. 0.1 mL isopropanol with 0.04 N HCl was added to each well to dissolve the purple formazan crystals. The optical density (OD) was measured on an ELISA plate reader with a test wavelength of 570 nm and a reference wavelength of 630 nm. Data of 3 replicates were averaged and normalized to indicated controls.
