# Supplementary material for: Maf1 suppression of ATF5-dependent mitochondrial unfolded protein response contributes to rapamycin-induced radio-sensitivity in lung cancer cell line A549
Source: Aging (Albany NY). 2021 Feb 26;13(5):7300–13. doi: 10.18632/aging.202584 (PMC7993702; doi:10.18632/aging.202584)
Supplement: Supplementary Figures [file aging-13-202584-s002.pdf]

SUPPLEMENTARY FIGURES

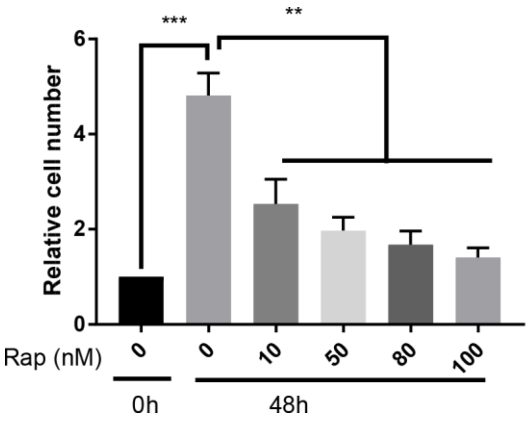

Supplementary Figure 1. Rapamycin reduced A549 proliferation in a dose-dependent manner. Cells were treated indicated rapamycin concentrations for 48 hours. Non-treated cells and cell at the time of plating (0h) were used as control. MTT assay was used to examine relative cell number and the colorimetric reads were normalized to non-treated cells at the time of plating. Error bars stands for 3 biological replicates. Mean difference was tested by t-test. **P<0.01, ***P<0.001.

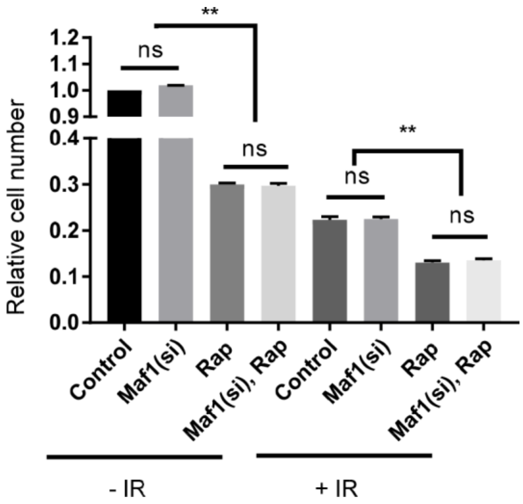

Supplementary Figure 2. Maf1 is not involved in rapamycin regulation of cell proliferation. Maf1 was knocked down by siRNA (si) in A549 cells. Control and knockdown cells were then irradiated (IR) with 6 Gy x-ray and treated with 100 nM rapamycin (Rap) as indicated. MTT assay was used to examine relative cell number and the colorimetric reads were normalized to non-treated cells. Error bars stands for 3 technical replicates. Mean difference was tested by t-test. ns, not significant, **P<0.01.
